# Supplementary material for: Predictors of prior HIV testing and acceptance of a community-based HIV test offer among male bar patrons in northern Tanzania
Source: PLOS Glob Public Health. 2024 Feb 26;4(2):e0002946. doi: 10.1371/journal.pgph.0002946 (PMC10896543; doi:10.1371/journal.pgph.0002946)
Supplement: Attachment — Submitted filename: Response to Reviewers.docx [file pgph.0002946.s005.docx]

**Reviewer #1**

1. *Comment*: This is an interesting and well-written paper that could be published and I don’t have further comments or questions overall. I was however interested in the outcomes for the 17 people who tested HIV positive and whose lives will have been significantly changed from helping with a research survey.

*Response*: We facilitated linkage to care for all participants who were newly diagnosed with HIV. Specifically, participants were provided with choices of Care and Treatment Centers (CTC) in Moshi, and study personnel offered to meet each participant at the CTC of their choice to facilitate the enrollment process. Unfortunately, we did not track linkage to care or their outcomes. We have added this clarification in the revised manuscript (see lines 134-135)

**Reviewer #2**

1. *Comment*: The authors write about factors associated with prior and current HIV testing uptake. However, it is not clear why the authors chose to concentrate on both prior and current HIV testing uptake instead of concentrating on one of the two areas. In my view, a focus on factors associated with current HIV testing uptake would suffice with no need to examine factors associated with prior HIV testing uptake. If the authors insist on including both areas, the authors should justify why it is crucial to examine both areas given the current trends in HIV testing behaviors among men.

*Response*: This secondary analysis intends to evaluate if individual-level characteristics that predict prior HIV test-seeking are also associated with the decision to accept an HIV test offer. For this reason, we first evaluated the individual-level characteristics associated with prior HIV testing and then evaluated those associated with accepting a testing offer. We found that individual-level characteristics associated with prior HIV testing differed from those associated with accepting an HIV test offer. Taken together, our results highlight that men are willing to test for HIV, and efforts to expand the reach of testing are likely to increase HIV testing coverage among men. We have made edits to the Abstract, Background, and Discussion to emphasize the main message of our manuscript.

2. *Comment*: This manuscript focuses on community-based HIV testing. However, in the entire manuscript, I did not see any description of community-based HIV testing. What I saw was that men were approached in the bars and they were asked to come to the research office for eligibility screening. If eligible, men were interviewed at the same place of screening. So, where is the community-based HIV testing referred to, which forms the main gist of the paper? In response, I would recommend that the authors provide a clear description of the community-based HIV testing offered, and how the issue of recruiting men from bars and interviewing them at the research office fits into this arrangement.

*Response*: Community-based HIV testing can be defined as testing that is performed outside of a health facility. In this study, men were recruited from bars and then received HIV testing on a day different from that of recruitment at an office that was not connected or associated with a facility-based testing center. We have added this clarification in the Methods (please see lines 91-94). In our original manuscript (see citation 12), we highlight the importance of future studies evaluating the feasibility of on-site testing at bars.

3. *Comment*: On page 11, the authors write, “Among those who accepted testing, 17 (5.4%) were found to be HIV-infected. We detected 1 (1.3%) infection among participants who reported testing ≤ 12 months prior, 10 (6.8%) among those who tested > 12 months prior, and 6 (6.3%) among never-testers”. I could not trace where the authors picked this information from. If it was published in a previous paper, then only reference can be made to that paper but no results should be reproduced as if these findings were part of the current analysis.

*Response*: Our survey collected self-reported HIV testing history from all participants. This is clarified in our revised manuscript (see lines 119-120). The categories of HIV testing (≤ 12 months vs. > 12 months vs. never-tested) as reported in this manuscript differ from those presented in our prior manuscript (≤ 12 months vs. > 12 months).

4. *Comment*: The authors should describe the context in the bars that led the study team to consider them as study sites. This information would help the readers to appreciate why the study team opted to recruit men from bars rather than from other places.

*Response*: Please see changes to the Background (lines 59-62) and Discussion (lines 235-238).

5. *Comment*: The authors indicate that all the men at the bars were approached and invited for interviews with the exception of those that were intoxicated. However, interviews were conducted on a day different from the recruitment day. If this was the case, then, why would they not invite every man at the bars to come to the screening site – since they would not be intoxicated at the time of interview? I would understand the issue of leaving out intoxicated men if the interviews were to be conducted at the bar, immediately after being selected.

*Response*: The reviewer highlights an important point that was discussed extensively by our research team during the planning of our study. Our decision to not approach intoxicated men was driven by two considerations. First, we anticipated that intoxicated men would not have the capacity to understand the context of the study as explained by our research personnel during recruitment. Second, the decision to not approach intoxicated men was in part due to safety concerns for our research personnel.

6. *Comment*: Although the authors refer to “predictors of prior HIV testing”, I did not see any description of what constituted “prior HIV testing”. How was prior HIV testing defined and measured? There was no indication that any data on prior HIV testing were collected.

Response: Prior testing history was self-reported. Please see the changes in lines 121-122.

7. *Comment*: Please include the study approval numbers from the Research and Ethics Committees that approved the study.

*Response*: Please see the addition of approval numbers in the sub-section Ethical Consideration. Moreover, approval documents are included in the submission as required by *PLOS Global Public Health*.

8. *Comment*: On page 10, the authors present the “baseline characteristics of the cohort”. I could not understand what “baseline” visit that the authors referred to since there was no prior mention of baseline or follow-up visits before getting to page 10; and there is also no mention of any cohort that was followed up as part of the study. If this analysis is based on data collected as part of a population-based cohort, then, there should be a description of the cohort from which data were drawn as part of the ‘Methods’ section. But even then, I would not refer to ‘baseline’ unless there is a particular reason for doing so.

*Response*: This was a cross-sectional study. All references to “baseline” are now removed.

9. *Comment*: In Table 1, the authors refer to ‘Response to HIV testing offer’ as a correlate under the “HIV testing” characteristic but also indicate a possible cross-tabulation with ‘Response to HIV test offer’. In one way or the other, there is duplication here that can be avoided. To understand what I mean, the authors can re-examine how “response to HIV testing offer” (coded as “declined/accepted”) was cross-tabulated with “response to HIV test offer” (coded as “declined/accepted”). One of the two aspects would have to be dropped.

*Response*: We dropped the row titled ‘Response to HIV testing offer’ in Table 1.

10. *Comment*: The word “HIV infected” should be replaced. It is no longer in use as it is considered stigmatizing.

*Response*: We removed the terminology.

11. *Comment*: I did not see any attempt to characterize the men interviewed as part of the study as “bar patrons”, other than the fact that this is mentioned in the paper. How can we be sure that the men interviewed were bar patrons? This is important given that the findings reported in the paper can also be reported from any other paper that targeted men in the general population. Also, any failure to confirm that the study participants were indeed male patrons in a bar setting affects the generalizability of the findings. I think more information can be provided about the men that were recruited into the study, to characterize them as bar patrons, different from men picked from the general population.

*Response*: We have added additional text in the methods for clarification. Briefly, eligible male bar patrons were provided a recruitment card that was uniquely numbered, dated, and valid for one month. Individuals who presented to our study office without a valid recruitment card were not eligible to participate in the study. Please see the changes in lines 89-98.

12. *Comment*: Finally, in the discussion section, the bar setting should be one of the issues discussed. This should begin by identifying bars as places where risk behavior occurs, and then describing male patrons as an HIV-risk group. The discussion can then continue to focus on why recruiting men from such setting was necessary and the implications of the findings on research and HIV prevention efforts in similar settings.

*Response*: We agree with the reviewer that this concept requires further emphasis. Our previous manuscript details the importance of alcohol-serving establishments as venues frequented by men at risk for HIV. Please see the changes in the Background (lines 59-62).
