# Supplementary material for: Predictors of prior HIV testing and acceptance of a community-based HIV test offer among male bar patrons in northern Tanzania
Source: PLOS Glob Public Health. 2024 Feb 26;4(2):e0002946. doi: 10.1371/journal.pgph.0002946 (PMC10896543; doi:10.1371/journal.pgph.0002946)
Supplement: Attachment — Submitted filename: Response to Reviewers.docx [file pgph.0002946.s006.docx]

1. *Comments*: There were few minor numerical inconsistencies I have noticed which I think may need to be addressed for the sake uniformity.

- For instance, in Line 182 on page 9, the 15 (39.4%) was a little different from that in the table 2 as 15 (39.5%) which were both rounded to one decimal place. Since the real ratio is 15/38=39.47, I think it would be better to keep it 39.5% to one decimal place to make the in-text figures consistent with the figures in the tables.
- Same applies to Line 39 on page 2 under Abstract, where adjusted Prevalence Ratios and 95%CI ……HIV knowledge (1.05, 1.02-1.08), which was a little different from line 194 on page 10……HIV knowledge (1.04, 1.01-1.07).

*Response*: We have made the change to 39.5% throughout. Regarding the adjusted prevalence ratios, the prevalence ratio with 95% CI has been corrected in the abstract to reflect the adjusted prevalence ratio.

2. Comment: (Table 3) I am wondering why the authors decided to combine married, divorced and widowed together. I am not sure if their willingness to take risk or risky behavior will be same. Maybe the authors could consider segregating this and use married as the reference.

*Response*: Marital, divorced, and widowed were combined since marriage or prior marriage is known as one predictor of prior HIV testing among men. We evaluated different constructions of this variable and did not see an association with prior testing.

3. *Comment*: (Discussion) I suggest that this should rather be discussion and conclusion since the last paragraph concludes the study.

Response: We have included a Conclusion section header.
